# Supplementary material for: Pyruvate Dehydrogenase Kinase 1 inhibition mediated oxidative phosphorylation enhancement in cartilage promotes osteoarthritis progression
Source: BMC Musculoskelet Disord. 2023 Jul 20;24:597. doi: 10.1186/s12891-023-06585-6 (PMC10357736; doi:10.1186/s12891-023-06585-6)
Supplement: Supplementary file 1 — Additional file 1. [file 12891_2023_6585_MOESM1_ESM.zip › BP.Gsea.1653623667859/GOBP_SKELETAL_MUSCLE_CELL_DIFFERENTIATION.html]

Details for gene set GOBP\_SKELETAL\_MUSCLE\_CELL\_DIFFERENTIATION[GSEA]

|  || Dataset | 55457\_collapsed\_to\_symbols.cls.cls #OA\_versus\_normal.cls.cls #OA\_versus\_normal\_repos |
| Phenotype | cls.cls#OA\_versus\_normal\_repos |
| Upregulated in class | normal |
| GeneSet | GOBP\_SKELETAL\_MUSCLE\_CELL\_DIFFERENTIATION |
| Enrichment Score (ES) | -0.48846003 |
| Normalized Enrichment Score (NES) | -1.7893858 |
| Nominal p-value | 0.0 |
| FDR q-value | 0.10853936 |
| FWER p-Value | 0.983 |
Table: GSEA Results Summary

  

Fig 1: Enrichment plot: GOBP\_SKELETAL\_MUSCLE\_CELL\_DIFFERENTIATION      
 Profile of the Running ES Score & Positions of GeneSet Members on the Rank Ordered List

  

| SYMBOL | TITLE | RANK IN GENE LIST | RANK METRIC SCORE | RUNNING ES | CORE ENRICHMENT || 1 | MSTN | myostatin [Source:HGNC Symbol;Acc:HGNC:4223] | 723 | 0.588 | -0.0190 | No |
| 2 | HIVEP3 | HIVEP zinc finger 3 [Source:HGNC Symbol;Acc:HGNC:13561] | 1030 | 0.511 | -0.0105 | No |
| 3 | HLF | "HLF transcription factor, PAR bZIP family member [Source:HGNC Symbol;Acc:HGNC:4977]" | 1461 | 0.434 | -0.0164 | No |
| 4 | SIX1 | SIX homeobox 1 [Source:HGNC Symbol;Acc:HGNC:10887] | 2011 | 0.360 | -0.0363 | No |
| 5 | KLHL41 | kelch like family member 41 [Source:HGNC Symbol;Acc:HGNC:16905] | 2374 | 0.318 | -0.0444 | No |
| 6 | PAX5 | paired box 5 [Source:HGNC Symbol;Acc:HGNC:8619] | 2546 | 0.298 | -0.0389 | No |
| 7 | EMD | emerin [Source:HGNC Symbol;Acc:HGNC:3331] | 3404 | 0.211 | -0.0922 | No |
| 8 | MYF6 | myogenic factor 6 [Source:HGNC Symbol;Acc:HGNC:7566] | 4057 | 0.150 | -0.1334 | No |
| 9 | MEF2C | myocyte enhancer factor 2C [Source:HGNC Symbol;Acc:HGNC:6996] | 4218 | 0.135 | -0.1373 | No |
| 10 | COPS2 | COP9 signalosome subunit 2 [Source:HGNC Symbol;Acc:HGNC:30747] | 4494 | 0.113 | -0.1515 | No |
| 11 | TBX1 | T-box transcription factor 1 [Source:HGNC Symbol;Acc:HGNC:11592] | 4679 | 0.098 | -0.1597 | No |
| 12 | HEYL | hes related family bHLH transcription factor with YRPW motif like [Source:HGNC Symbol;Acc:HGNC:4882] | 4820 | 0.086 | -0.1651 | No |
| 13 | SAP30 | Sin3A associated protein 30 [Source:HGNC Symbol;Acc:HGNC:10532] | 4907 | 0.079 | -0.1668 | No |
| 14 | NUPR1 | "nuclear protein 1, transcriptional regulator [Source:HGNC Symbol;Acc:HGNC:29990]" | 5616 | 0.023 | -0.2204 | No |
| 15 | SOX11 | SRY-box transcription factor 11 [Source:HGNC Symbol;Acc:HGNC:11191] | 5822 | 0.007 | -0.2359 | No |
| 16 | RB1 | RB transcriptional corepressor 1 [Source:HGNC Symbol;Acc:HGNC:9884] | 6013 | -0.006 | -0.2503 | No |
| 17 | RBFOX1 | RNA binding fox-1 homolog 1 [Source:HGNC Symbol;Acc:HGNC:18222] | 6152 | -0.016 | -0.2600 | No |
| 18 | ANKRD1 | ankyrin repeat domain 1 [Source:HGNC Symbol;Acc:HGNC:15819] | 6416 | -0.037 | -0.2782 | No |
| 19 | GTF3C5 | general transcription factor IIIC subunit 5 [Source:HGNC Symbol;Acc:HGNC:4668] | 6506 | -0.043 | -0.2824 | No |
| 20 | HMG20B | high mobility group 20B [Source:HGNC Symbol;Acc:HGNC:5002] | 6768 | -0.061 | -0.2989 | No |
| 21 | CITED2 | Cbp/p300 interacting transactivator with Glu/Asp rich carboxy-terminal domain 2 [Source:HGNC Symbol;Acc:HGNC:1987] | 7100 | -0.087 | -0.3191 | No |
| 22 | MYOD1 | myogenic differentiation 1 [Source:HGNC Symbol;Acc:HGNC:7611] | 7839 | -0.143 | -0.3674 | No |
| 23 | EPHB1 | EPH receptor B1 [Source:HGNC Symbol;Acc:HGNC:3392] | 8342 | -0.181 | -0.3950 | No |
| 24 | PHOX2B | paired like homeobox 2B [Source:HGNC Symbol;Acc:HGNC:9143] | 9179 | -0.246 | -0.4444 | No |
| 25 | MYF5 | myogenic factor 5 [Source:HGNC Symbol;Acc:HGNC:7565] | 9572 | -0.280 | -0.4572 | No |
| 26 | BCL9 | BCL9 transcription coactivator [Source:HGNC Symbol;Acc:HGNC:1008] | 9587 | -0.282 | -0.4404 | No |
| 27 | FOXN2 | forkhead box N2 [Source:HGNC Symbol;Acc:HGNC:5281] | 9736 | -0.295 | -0.4333 | No |
| 28 | NR1D2 | nuclear receptor subfamily 1 group D member 2 [Source:HGNC Symbol;Acc:HGNC:7963] | 9968 | -0.318 | -0.4311 | No |
| 29 | FOS | "Fos proto-oncogene, AP-1 transcription factor subunit [Source:HGNC Symbol;Acc:HGNC:3796]" | 10640 | -0.387 | -0.4588 | No |
| 30 | CYP26B1 | cytochrome P450 family 26 subfamily B member 1 [Source:HGNC Symbol;Acc:HGNC:20581] | 11022 | -0.433 | -0.4611 | Yes |
| 31 | GPC1 | glypican 1 [Source:HGNC Symbol;Acc:HGNC:4449] | 11096 | -0.443 | -0.4387 | Yes |
| 32 | MEF2D | myocyte enhancer factor 2D [Source:HGNC Symbol;Acc:HGNC:6997] | 11149 | -0.451 | -0.4142 | Yes |
| 33 | NR4A1 | nuclear receptor subfamily 4 group A member 1 [Source:HGNC Symbol;Acc:HGNC:7980] | 11709 | -0.535 | -0.4239 | Yes |
| 34 | DDX17 | DEAD-box helicase 17 [Source:HGNC Symbol;Acc:HGNC:2740] | 11716 | -0.535 | -0.3905 | Yes |
| 35 | BTG2 | BTG anti-proliferation factor 2 [Source:HGNC Symbol;Acc:HGNC:1131] | 11917 | -0.576 | -0.3696 | Yes |
| 36 | EGR1 | early growth response 1 [Source:HGNC Symbol;Acc:HGNC:3238] | 12052 | -0.607 | -0.3416 | Yes |
| 37 | ARNTL | aryl hydrocarbon receptor nuclear translocator like [Source:HGNC Symbol;Acc:HGNC:701] | 12120 | -0.625 | -0.3073 | Yes |
| 38 | MYOG | myogenin [Source:HGNC Symbol;Acc:HGNC:7612] | 12313 | -0.681 | -0.2791 | Yes |
| 39 | AKIRIN1 | akirin 1 [Source:HGNC Symbol;Acc:HGNC:25744] | 12407 | -0.709 | -0.2415 | Yes |
| 40 | NOTCH1 | notch receptor 1 [Source:HGNC Symbol;Acc:HGNC:7881] | 12555 | -0.776 | -0.2038 | Yes |
| 41 | ATF3 | activating transcription factor 3 [Source:HGNC Symbol;Acc:HGNC:785] | 12659 | -0.833 | -0.1591 | Yes |
| 42 | KLF5 | Kruppel like factor 5 [Source:HGNC Symbol;Acc:HGNC:6349] | 12672 | -0.845 | -0.1066 | Yes |
| 43 | MAFF | MAF bZIP transcription factor F [Source:HGNC Symbol;Acc:HGNC:6780] | 12766 | -0.925 | -0.0553 | Yes |
| 44 | DDX5 | DEAD-box helicase 5 [Source:HGNC Symbol;Acc:HGNC:2746] | 12834 | -1.038 | 0.0051 | Yes |
Table: GSEA details [plain text format]

  

Fig 2: GOBP\_SKELETAL\_MUSCLE\_CELL\_DIFFERENTIATION      
 Blue-Pink O' Gram in the Space of the Analyzed GeneSet

  

Fig 3: GOBP\_SKELETAL\_MUSCLE\_CELL\_DIFFERENTIATION: Random ES distribution      
 Gene set null distribution of ES for **GOBP\_SKELETAL\_MUSCLE\_CELL\_DIFFERENTIATION**
